# NMUR1 in the NMU-Mediated Regulation of Bone Remodeling

**DOI:** 10.3390/life11101028

**Published:** 2021-09-29

**Authors:** Yu-Tin Hsiao, Kelli J. Manikowski, Samantha Snyder, Nicole Griffin, Ashley L. Orr, Elizabeth Q. Hulsey, Gabriella Born-Evers, Tara Zukosky, Maria E. Squire, Julia M. Hum, Corinne E. Metzger, Matthew R. Allen, Jonathan W. Lowery

**Affiliations:** 1 Division of Biomedical Science, Marian University College of Osteopathic Medicine, Indianapolis, IN 46022, USA; yhsiao101@marian.edu (Y.-T.H.); kjestes091@marian.edu (K.J.M.); ssnyder383@marian.edu (S.S.); ngriffin860@marian.edu (N.G.); adaniel348@marian.edu (A.L.O.); ehulsey329@marian.edu (E.Q.H.); gborn502@marian.edu (G.B.-E.); jmhum@marian.edu (J.M.H.); 2 Bone and Muscle Research Group, Marian University, Indianapolis, IN 46022, USA; 3 Department of Biology, The University of Scranton, Scranton, PA 18503, USA; tzukosky@student.touro.edu (T.Z.); maria.squire@scranton.edu (M.E.S.); 4 Department of Anatomy, Cell Biology and Physiology, Indiana University School of Medicine, Indianapolis, IN 46202, USA; cormetzg@iu.edu (C.E.M.); matallen@iu.edu (M.R.A.); 5 Indiana Center for Musculoskeletal Health, Indiana University School of Medicine, Indianapolis, IN 46202, USA

**Keywords:** Neuromedin-U, NMU, NMUR1, NMUR2, bone, osteoblast, osteoporosis

## Abstract

Neuromedin-U (NMU) is an evolutionarily conserved peptide that regulates varying physiologic effects including blood pressure, stress and allergic responses, metabolic and feeding behavior, pain perception, and neuroendocrine functions. Recently, several lines of investigation implicate NMU in regulating bone remodeling. For instance, global loss of NMU expression in male and female mice leads to high bone mass due to elevated bone formation rate with no alteration in bone resorption rate or observable defect in skeletal patterning. Additionally, NMU treatment regulates the activity of osteoblasts in vitro. The downstream pathway utilized by NMU to carry out these effects is unknown as NMU signals via two G-protein-coupled receptors (GPCRs), NMU receptor 1 (NMUR1), and NMU receptor 2 (NMUR2), and both are expressed in the postnatal skeleton. Here, we sought to address this open question and build a better understanding of the downstream pathway utilized by NMU. Our approach involved the knockdown of *Nmur1* in MC3T3-E1 cells in vitro and a global knockout of *Nmur1* in vivo. We detail specific cell signaling events (e.g., mTOR phosphorylation) that are deficient in the absence of NMUR1 expression yet trabecular bone volume in femora and tibiae of 12-week-old male *Nmur1* knockout mice are unchanged, compared to controls. These results suggest that NMUR1 is required for NMU-dependent signaling in MC3T3-E1 cells, but it is not required for the NMU-mediated effects on bone remodeling in vivo. Future studies examining the role of NMUR2 are required to determine the downstream pathway utilized by NMU to regulate bone remodeling in vivo.

## 1. Introduction

In humans, bone mass generally declines after the third decade of life due to the rate of bone resorption, carried out by osteoclasts, exceeding the rate of bone formation, carried out by osteoblasts [1]. Osteoporosis, characterized by low bone mass, places individuals at greater risk for fracture, disability, and death [2]. In the US, hospitalizations for osteoporotic fractures exceed those for heart attack, stroke, and breast cancer combined [3]. Osteoporosis rates are expected to rise significantly in the coming decades, with an estimated 3 million osteoporotic fractures per year by 2025 in the US [4,5]. However, there are limited pharmacological treatment options for osteoporosis, particularly for the long-term management of this chronic condition. A more complete understanding of the molecular pathways regulating the balance of bone resorption and bone formation may reveal new therapeutic approaches for improving bone mass and decreasing fracture risk in patients.

Neuromedin-U (NMU) is an evolutionarily conserved peptide with multiple physiologic effects including blood pressure regulation, stress, and allergic responses, metabolic and feeding behavior, pain perception, neuroendocrine functions, and the ability to induce smooth muscle contraction in a variety of organs [6,7,8]. In addition, two independent studies implicate NMU in regulating bone remodeling in vivo. For instance, Sato et al. reported that global loss of NMU expression in male and female mice leads to high bone mass by 12 weeks of age due to elevated bone formation rate with no alteration in bone resorption rate or observable defect in skeletal patterning [9]. Hsiao et al. corroborated the high trabecular bone mass phenotype in global *Nmu* mutant mice [10].

NMU is ubiquitously distributed in two major molecular forms: a 25-amino-acid (a.a.) peptide (NMU25) and an 8 a.a. peptide (NMU8) [11]. NMU8 is derived from the C-terminus of NMU25 and both isoforms display similar receptor affinity in vitro for the heterotrimeric Gq/11-protein-coupled receptors NMU receptor 1 (NMUR1) and NMU receptor 2 (NMUR2) [11]. NMUR1 is more broadly expressed than NMUR2 (see the Human Protein Atlas, proteinatlas.org, accessed on 26 September 2021), with NMUR1 generally expressed in peripheral tissues and NMUR2 expressed predominantly in the central nervous system [12]. That said, mRNA for both receptors, as well as *Nmu*, are readily detectable in the postnatal bone microenvironment and in osteogenic bone marrow stromal cells and MC3T3-E1 osteoblast-like cells [10]. It is unknown, however, which receptor is utilized by NMU to carry out its effects on bone cells in vivo.

Here, we sought to build a better understanding of the downstream pathway utilized by NMU. Our approach involved the knockdown of *Nmur1* in MC3T3-E1 cells in vitro and a global knockout of *Nmur1* in vivo. We detail specific cell signaling events (e.g., mTOR phosphorylation) that are deficient in the absence of NMUR1 expression, yet bone mass is unchanged in *Nmur1* knockout mice, compared to controls.

## 2. Materials and Methods

### 2.1. Cells

MC3T3-E1 subclone 4 cells were acquired from ATCC (Manassas, VA, USA) and routinely cultured in a growth medium (MEM-alpha without ascorbic acid (Thermo Fisher Scientific, Waltham, MA, USA) supplemented with 10% fetal bovine serum (FBS) (Sigma-Aldrich, St. Louis, MO, USA)) at 37 °C in a humidified incubator with an atmosphere of 5% CO_2_. For transductions, MC3T3-E1 cells were seeded into 96-well plates at a density of 1.6 × 10^4^ cells per well in the growth medium. The cells were incubated overnight then the growth medium was aspirated and replaced with a new growth medium containing 8 µg/mL hexadimethrine bromide (Sigma Aldrich) after which the plate was gently swirled to mix. Replication-incompetent lentiviral particles from Sigma-Aldrich (anti-*Nmur1*: clone 1 TRCN0000004644, clone 2 TRCN0000004643, clone 3 TRCN00000420683, clone 4 TRCN00000418924, clone 5 TRCN0000010899; pLKO.1-puro-CMV-TurboGFP positive control, or pLKO.1-puro non-mammalian shRNA control) were then added to specific wells with the volume determined by the viral particle concentration using a multiplicity of infection of three. The cells were incubated overnight before the media was aspirated, washed with 1× PBS (Caisson Labs, Smithfield, UT, USA), and cultured in a selection medium (growth medium containing 2 µg/mL puromycin (AG Scientific P-1033)). The parental lines were cultured separately in a selection medium with media replacement every 3–4 days; no attempt was made to generate single-cell clones in order to account for the possibility of site-specific integration effects on cell behavior.

For signaling studies, cells were seeded at 1 × 10^6^ per well in 6 cm dishes in a selection medium. After 24 h, the media was exchanged to MEM-alpha without ascorbic acid (Thermo Fisher, Waltham, MA, USA) supplemented with 0.5% FBS (Sigma-Aldrich, St. Louis, MO, USA). After 24 h, some wells were supplemented with 1 μM NMU25 (Bachem, Bubendorf, Switzerland) for four hours, and then the cells were collected for analysis.

### 2.2. Gene Expression Studies

Total RNA was collected from cell cultures using an RNEasy Plus Kit (QIAGEN, Hidden, Germany). Reverse transcriptase was performed using the SuperScript III First-Strand Synthesis SuperMix (Thermo Fisher) with oligo dT according to the manufacturer’s instructions. Quantification of target gene expression was performed using TaqMan Gene Expression Assays (Applied Biosystems) on a QuantStudio 3 instrument (Thermo Fisher) with the following probes: *Bglap*, Mm03413826_mH; *Nmu*, Mm00479868_m1; *Nmur1*, Mm00515885_m1; *Runx2*, Mm00501584_m1; *Sp7*, Mm_04933803_m1; and *Gapdh*, Mm99999915_g1. The reaction solution was prepared by mixing the synthesized cDNA with TaqMan Universal PCR Master Mix (Thermo Fisher), the specific probe, and water as directed by the manufacturer. Data were analyzed via the 2^−ΔΔCT^ method relative to *Gapdh*.

### 2.3. Antibody Array

For protein isolation, bones or cells were lysed in RIPA Buffer (Cell Signaling Technologies, Danvers, MA, USA) containing Halt Protease and Phosphatase Inhibitors (Thermo Fisher) according to the manufacturer’s instruction. Prior to lysis, frozen tibiae were cleaned of soft tissue, opened to expose the medullary cavity, centrifuged at 500× *g* for 2 min to separate marrow, and then homogenized using a Bullet Blender (Next Advance, Averill Park, NY, USA). Concentrations were determined using a BCA Assay Kit (Thermo Fisher) on a FluoSTAR (BMG LABTECH, Ortenberg, Germany) instrument. Lysates (15 µg total protein) were analyzed using the TGF-beta Phospho Antibody Array (Full Moon Biosystems, Sunnyvale, CA, USA) or the Phospho Explorer Antibody Array (Full Moon Biosystems) for tibiae or cell samples, respectively, according to the manufacturer’s directions using Cy3-streptavidin (Thermo Fisher), with the following modification: incubations were carried out at 4 °C in the protein labeling and coupling steps, and 1× HALT Protease and Phosphatase Inhibitors (ThermoFisher) was added in the protein labeling step. Signal intensity was determined by Full Moon Biosystems on a GenePix 4000B Imager (Molecular Devices, San Jose, CA, USA) using GenePix Pro software (Molecular Devices, San Jose, CA, USA) by an objective scorer blinded to sample identity. Results were normalized against GAPDH or total protein isoform as indicated in the text.

### 2.4. Mice

The following were received as a generous gift from David Artis (Weill Medical College, Cornell University, Ithaca, NY, USA): snap-frozen tibiae from 12-week-old female *Nmu* knockout mice [8] and controls; formalin-fixed right hindlimbs and sera from 12-week-old male *Nmur1* knockout mice [13]. Mice were fasted six hours prior to sacrifice, and sera were isolated as previously described [14]. Hindlimbs were fixed in 10% neutral buffered formalin, stored in 70% ethanol at 4 °C, and analyzed as detailed below. All animal procedures were in accordance with the Institutional Animal Care and Use Committee of Weill Medical College (protocol #2014-0032).

### 2.5. Micro Computed Tomography (µCT)

Tibiae and femora from *Nmur1* knockout and control mice were scanned using a high-resolution (10 μm/voxel) μCT 80 scanner (Scanco, Wayne, PA, USA). Bone volume fraction and other morphometric parameters were assessed for cortical bone in the mid-diaphyseal region (420 μm) of each bone and for trabecular bone in the proximal and distal metaphyseal regions of the tibia (800 μm) and femur (2320 μm) according to established guidelines [15]. A Gaussian filter was used to minimize noise and a global threshold distinguished bone from non-bone. Contour line drawing was performed by an objective scorer blinded to sample genotype and parameters were calculated using scanner-specific software.

### 2.6. Histological Analyses

Distal femora were serially dehydrated and embedded in methyl methacrylate (Sigma-Aldrich, St. Louis, MO, USA). Serial frontal sections were cut 4 μm thick and stained with von Kossa stain and MacNeal counterstain or with TRAP stain to identify osteoclasts. Histomorphometric analyses were completed using BIOQUANT (BIOQUANT Image Analysis, Nashville, TN, USA). A standard region of interest of the distal femur trabecular bone was utilized excluding primary spongiosa and endocortical surfaces. Osteoid surface (OS), osteoclast surface (OcS), and osteoclast number (OcN) normalized to bone surface (BS) were measured at 40× magnification within the region of interest. Measurements and analyses were performed using standardized guidelines [16] by an objective scorer blinded to sample genotype.

### 2.7. Serum Analysis

Serum levels of procollagen type I intact N-terminal propeptide (PINP) were measured using the Mouse PINP ELISA Kit by NeoScientific (Cambridge, MA, USA) according to the manufacturer’s instructions.

### 2.8. Statistical Analyses

Data were plotted in GraphPad Prism 8 (San Diego, CA, USA) and statistical significance was determined using unpaired *t*-test, unpaired *t*-test with Welch’s correction, unpaired to test with Bonferroni correction, ANOVA with Tukey correction, or ANOVA with Bonferroni correction as detailed in the figure or table legend; *p* < 0.05 was considered significant.

## 3. Results

### 3.1. Signaling Changes in NMU Knockout Bones

**Figure 1:**
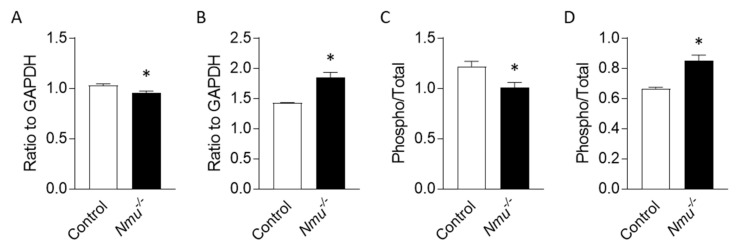
Antibody array analyses using tibiae from wild-type controls or Nmu knockout mice. Levels of TGF-alpha (**A**), PKC-theta phosphorylated at Threonine-538 (**B**), Shc phosphorylated at Tyrosine-427, and mTOR phosphorylated at Serine-2448. Data are presented as mean +/− SEM from n = 3 per genotype and relative to GAPDH (**A**,**B**) or phospho isoform relative to total (**C**,**D**). The full data set may be found in Supplementary Materials. * indicates *p* < 0.05 against control.

To investigate the downstream signaling pathway regulated by NMU in bone, we performed phospho-profiling antibody arrays on tibiae obtained from wild-type and global *Nmu* knockout mice. This revealed that of the 175 targets examined, the expression level or phosphorylation status relative to GAPDH housekeeping control (Table S1 in the supplementary) differed for only two factors between genotypes: the level of transforming growth factor (TGF)-alpha was reduced, while the level of protein kinase c-theta (PKC-theta) phosphorylated at Threonine-538 was increased in *Nmu* knockout tibiae, compared to wild-type controls (Figure 1A,B). Two additional factors differed in the ratio of the phosphorylated isoform relative to that target’s total expression level: the level of Src homology and collagen adaptor protein (Shc) phosphorylated at Tyrosine-427 was increased, while the mechanistic target of rapamycin kinase (mTOR) phosphorylated at Serine-2448 was decreased in *Nmu* knockout tibiae, compared to wild-type controls (Figure 1C,D and Table S2).

### 3.2. NMU Regulates Osteoblast Function through NMUR1 In Vitro

**Figure 2:**
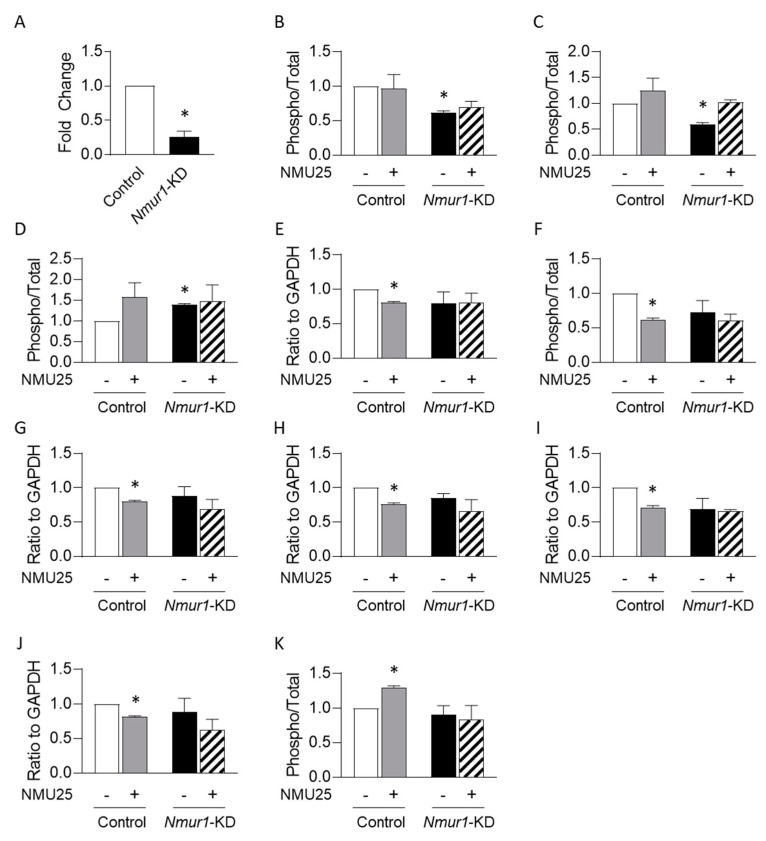
(**A**)**:** Expression of *Nmur1* in scrambled control cells and *Nmur1* knockdown lines (*Nmur1*-KD) by quantitative RT-PCR. Data are presented as mean +/− SEM normalized to scrambled control from n = 3 biological replicates per cell line. * indicates *p* < 0.05 against scrambled control by paired *t*-test; (**B**–**K**)**:** antibody array analyses using lysates from scrambled control or *Nmur1*-KD cells +/− treatment with 1 µM NMU25 for four hours. Levels of MEF2C phosphorylated at Serine-396 (**B**), Raf1 phosphorylated at Serine-259 (**C**), NFkB-p65 phosphorylated at Serine-468 (**D**), total DNA-PK (**E**), BRCA1 phosphorylated at Serine-1457 (**F**), EGFR phosphorylated at Threonine-693 (**G**), Rb phosphorylated at Threonine-821 (**H**), Src phosphorylated at Tyrosine-529 (**I**), WEE1 phosphorylated at Serine-642 (**J**), and mTOR phosphorylated at Serine-2448 (**K**). Data are presented as mean +/− SEM of the target relative to GAPDH (**E**–**J**) or phospho isoform relative to total (**B**–**D**,**K**) from n = 3 biological replicates per cell line per condition. The full data set may be found in Tables S3 and S4. * indicates *p* < 0.05 against scrambled control by repeated-measures ANOVA with Tukey correction.

Having identified putative downstream signaling effectors of the NMU pathway in bone, we next sought to examine the receptor utilized by NMU in bone cells. We previously demonstrated that the murine osteoblastic cell line MC3T3-E1 responds to exogenous NMU treatment and expresses NMUR1 [10]. Thus, we utilized lentiviral-mediated delivery of short-hairpin RNA (shRNA) to establish MC3T3-E1 cells in which *Nmur1* expression was knocked down. Transduction conditions were first established using lentivirus carrying cDNA encoding *Green Fluorescent Protein* (*Gfp*), which revealed that a multiplicity of infection of three resulted in robust expression of GFP by 72 h post transduction (data not shown). We then utilized lentivirus for delivery of five different shRNA clones (or scrambled control) and, after puromycin selection, analyzed the efficiency of *Nmur1* knockdown by qRT-PCR. This revealed that shRNA clone 3 resulted in greater than 70% knockdown of *Nmur1* levels, compared to scrambled control cells (Figure 2A), whereas the other clones were markedly less effective (Figure S1 in the supplementary).

To provide functional evidence for the downstream signaling pathway regulated by NMU in MC3T3-E1 cells, we performed phospho-profiling antibody arrays on scrambled control and *Nmur1*-KD cells. Under untreated conditions, *Nmur1*-KD cells displayed no differences compared to scrambled controls in the expression level or phosphorylation status of any of the 1317 targets relative to GAPDH housekeeping control (Table S3). A few targets, however, differed between the *Nmur1*-KD and scrambled control cell lines in their phosphorylation status relative to the total amount of that target (Table S4). For instance, under untreated conditions, Myocyte enhancer factor 2c (MEF2C) phosphorylated at Serine-396 and RAF proto-oncogene serine/threonine-protein kinase (Raf1) phosphorylated at Serine-259 were reduced relative to total MEF2C or Raf1 levels, respectively, in *Nmur1*-KD cells compared to scrambled controls (Figure 2B,C). Conversely, *Nmur1*-KD cells displayed increased levels of phosphorylated NFkB-p65 at Serine-468 relative to total NFkB-p65 levels (Figure 2D). Since MC3T3-E1 cells express *Nmu* [10], this suggests that these factors may be regulated by NMU signaling endogenously. However, the biological relevance of these findings is uncertain, as the expression of osteoblastic markers *Runx2*, *Sp7/Osterix*, and *Bglap/Osteocalcin* do not differ between scrambled and *Nmur1*-KD MC3T3-E1 cells (Figure S2A–C).

Exposure of scrambled control cells to exogenous NMU25 for four hours led to changes in the expression level or phosphorylation status of several targets (Tables S3 and S4). In particular, in scrambled control cells, NMU25 treatment reduced the expression of DNA-dependent protein kinase catalytic subunit (DNA-PK) and the phosphorylation of Breast cancer type 1 susceptibility protein (BRCA1) at Serine-1457, Epidermal growth factor receptor (EGFR) at Threonine-693, Retinoblastoma-associated protein (Rb) at Threonine-821, Proto-oncogene tyrosine-protein kinase Src (Src) at Tyrosine-529, and WEE1 G2 checkpoint kinase (WEE1) at Serine-642 relative to GAPDH housekeeping control (Figure 2E–J). Each of these responses was absent in *Nmur1*-KD cells (Figure 2E–J). Additionally, in scrambled control cells, NMU25 treatment led to increased phosphorylation of mTOR at Serine-2448 relative to total mTOR levels (Figure 2K). These responses, however, were absent in *Nmur1*-KD cells (Figure 2E,F), consistent with our hypothesis that NMU signals via NMUR1 in osteoblastic cells.

### 3.3. Bone Volume Is Unchanged in the Absence of NMUR1

To examine the functional role of NMUR1 in the NMU-mediated regulation of bone remodeling in vivo, we performed µCT analyses on the distal and proximal metaphyseal region of the femora and tibiae, respectively, of wild-type and *Nmur1* knockout mice. At both sites, the trabecular bone volume fraction (BV/TV) in *Nmur1* knockout mice is comparable to controls (Table 1). Consistent with this, there are no observable differences in trabecular number (Tb.N), trabecular thickness (Tb.Th), trabecular separation (Tb.Sp), trabecular connectivity density (Conn.D), or structural model index (SMI) in *Nmur1* knockout mice (Table 1).

We also performed µCT on the mid-diaphyseal region of femora and tibiae from wild-type and *Nmur1* knockout mice. Cortical bone volume fraction (BV/TV) is unchanged in the absence of NMUR1 (Table 2). Similarly, cortical thickness (Ct.Th) is comparable between wild-type and *Nmur1* knockout mice (Table 2).

### 3.4. Histological and Serum Analyses of NMUR1-Deficient Mice

Histomorphometric analyses on the distal metaphyseal region of femora from wild-type and *Nmur1* knockout mice (Figure S3) revealed that osteoclast surface (OcS/BS) and osteoclast number (OcN/BS) were unchanged in *Nmur1* knockout mice (Table 3), which is consistent with the idea that bone resorption rate is normal in the absence of NMUR1. Osteoid surface, while higher in the *Nmur1* knockout cohort, was not statistically different from controls (Table 3). Similarly, serum levels of the bone formation marker PINP were unchanged in *Nmur1* knockout mice (Control: 3561.43 ± 103.50 pg/mL; *Nmur1* knockouts: 3812.75 ± 165.77 pg/mL; n = 4 per genotype; *p* = 0.25 by unpaired *t*-test with Welch’s correction). Collectively, these data support the idea that bone formation and bone resorption rates are unaffected by the absence of NMUR1.

## 4. Discussion

We sought to establish the molecular pathway utilized by NMU in the regulation of bone remodeling [9,10]. Using high-throughput antibody arrays on tibiae from *Nmu* knockout mice, we determined that global loss of NMU expression is associated with relatively minimal signaling changes: *Nmu* knockout mice displayed lower levels of TGF-alpha and reduced levels of activation-related phosphorylation of Shc (at Tyrosine-427) and mTOR (at Serine-2448). We are unaware of reports implicating these factors as regulated by NMU signaling, but notably, treatment of MC3T3-E1 cells with exogenous NMU25 led to increased phosphorylation of mTOR at Serine-2448 and this response was absent in *Nmur1*-KD cells. Several other factors were differentially regulated in *Nmur1*-KD cells, compared to controls, but, besides mTOR, none of those changes were reflected in *Nmu* knockout tibiae compared to controls. We did not perform signaling analyses examining if other NMU peptides besides NMU25 (such as NMU8) retained activity in *Nmur1*-KD cells; however, NMU8 and NMU25 display similar receptor affinity in vitro for NMUR1, and a prior study indicated that both isoforms regulate similar downstream targets [10,11].

Taken together, these findings provide further evidence that NMU is capable of exerting direct effects on bone cells. That said, it is important to note that currently available data do not definitively conclude whether NMU controls bone formation in vivo via direct actions on osteoblast lineage cells (as supported by this study, Hsiao et al. [10], and Rucinski et al. [17]) or indirectly through actions elsewhere such as the hypothalamus (as suggested by Sato et al. [9]). Hence, to examine the in vivo relevance of our findings, we allowed for both possibilities through a global knockout strategy for *Nmur1*, which is more broadly expressed than *Nmur2* [10,12]. We hypothesized that, if the actions of NMU on bone remodeling in vivo are carried out through NMUR1, then *Nmur1* knockout mice would display a similar high trabecular bone mass phenotype as *Nmu* knockout mice. However, this hypothesis was not supported, as the bone mass of the *Nmur1* knockout cohort, while somewhat higher, was not statistically different from controls. Similarly, histomorphometric analyses reveal that osteoid surface, an indicator of osteoblast activity, was higher in the *Nmur1* knockout cohort but not statistically different from controls. It is possible that our study was statistically underpowered to detect a variable or subtle phenotype in *Nmur1* knockout mice due to a limited sample size. That said, Grubb’s test did not identify any outliers in our data set, and power analyses indicate a sample size of 40 for each genotype would be necessary to detect high trabecular bone mass in tibiae of *Nmur1* knockouts (at 80% power and alpha at 0.05).

Thus, we conclude that the requirement of NMUR1 for NMU-mediated regulation of bone remodeling in vivo is minimal. Although the role of NMUR2 in bone physiology is entirely unknown, our data suggest two hypotheses for testing in future work: (1) the effects of NMU on bone remodeling in vivo are predominantly accomplished via NMUR2 and (2) the absence of NMUR1 is compensated by NMUR2. Both of these hypotheses are possibilities since NMU isoforms are capable of signaling through both NMUR1 and NMUR2. It is important to note that the current data set is unable to discriminate between these possibilities, and future experimentation is required to determine the precise downstream pathway utilized by NMU to regulate bone remodeling in vivo. Hence, we propose studies investigating the gross bone phenotype of global *Nmur2* knockout mice and, potentially, in global *Nmur1*/*Nmur2* double knockout mice as a means of distinguishing between these possibilities and delineating the receptor utilization of NMU in bone remodeling events. Unfortunately, while global *Nmur2* knockout mice have been generated [11], those animals are not available to us at this time. Additionally, follow-up studies using cell type-specific and/or lineage-specific knockouts or knockdowns for NMU pathway components would aid in distinguishing between the direct and indirect actions of NMU in skeletal homeostasis.

## Figures and Tables

**Table 1:** µCT analyses from trabecular regions of femora and tibiae from wild-type (n = 5) and *Nmur1* knockout mice (n = 7). Data are mean ± SEM. *p* values determined by unpaired *t*-test. TV, tissue volume. BV, bone volume. Tb.N, trabecular number. Tb.Th, trabecular thickness. Tb.Sp, trabecular separation. Conn.D, connectivity density. SMI, structure model index.

	Parameter	Wild Type	*Nmur1* Knockout	*p* Value
Distal Femora	TV (mm^3^)	4.639 ± 0.256	4.567 ± 0.108	0.778
	BV/TV (ratio)	0.154 ± 0.023	0.195 ± 0.026	0.289
	Tb.N (#/mm)	4.767 ±.1956	5.387 ± 0.317	0.165
	Tb.Th (mm)	0.049 ± 0.003	0.050 ± 0.001	0.893
	Tb.Sp (mm)	0.204 ± 0.009	0.179 ± 0.011	0.145
	Conn.D (#/mm^3^)	161.456 ± 18.937	228.086 ± 32.950	0.148
	SMI	2.201 ± 0.235	1.828 ± 0.205	0.262
Proximal Tibiae	TV (mm^3^)	1.580 ± 0.099	1.752 ± 0.078	0.198
	BV/TV (ratio)	0.195 ± 0.024	0.230 ± 0.021	0.314
	Tb.N (#/mm)	5.554 ± 0.161	6.051 ± 0.253	0.165
	Tb.Th (mm)	0.051 ± 0.002	0.053 ± 0.001	0.585
	Tb.Sp (mm)	0.168 ± 0.007	0.152 ± 0.007	0.169
	Conn.D (#/mm^3^)	176.637 ± 21.492	235.875 ± 22.430	0.096
	SMI	2.091 ± 0.198	1.873 ± 0.151	0.394

**Table 2:** µCT analyses from midshaft regions of femora and tibiae from wild-type (n = 5) and *Nmur1* knockout mice (n = 7). Data are mean +/− SEM. *p* values determined by unpaired *t*-test. TV, tissue volume. BV, bone volume. Ct.Th, cortical thickness.

	Parameter	Wild Type	*Nmur1* Knockout	*p* Value
Mid-diaphysis Femora	TV (mm^3^)	0.832 ± 0.045	0.820 ± 0.026	0.824
	BV/TV (ratio)	0.276 ± 0.021	0.263 ± 0.016	0.641
	Ct.Th (mm)	0.141 ± 0.007	0.133 ± 0.008	0.514
Mid-diaphysis Tibiae	TV (mm^3^)	0.477 ± 0.031	0.449 ± 0.029	0.539
	BV/TV (ratio)	0.256 ± 0.018	0.239 ± 0.010	0.404
	Ct.Th (mm)	0.192 ± 0.009	0.186 ± 0.009	0.635

**Table 3:** Histological analyses from the trabecular region of femora from wild-type (n = 5) and *Nmur1* knockout mice (n = 7). Data are mean +/− SEM. *p* values determined by unpaired *t*-test. OS, osteoid surface. BS, bone surface. OcS, osteoclast surface. OcN, osteoclast number.

Parameter	Wild Type	*Nmur1* Knockout	*p* Value
Osteoid Surface (OS/BS, ratio)	4.059 ± 1.361	8.400 ± 2.217	0.163
Osteoclast Surface (OcS/BS, ratio)	0.815 ± 0.309	0.608 ± 0.181	0.552
Osteoclast Number (OcN/BS, #/mm^2^)	0.036 ± 0.010	0.032 ± 0.007	0.764

## Data Availability

The datasets used and/or analyzed during the current study are available from the corresponding author on reasonable request.

## Supplementary Materials

The following are available online at www.mdpi.com/article/10.3390/life11101028/s1, Figure S1: Expression of *Nmur1* in scramble control cells and putative *Nmur1* knockdown (KD) lines; Figure S2: Expression of *Runx2* (A), *Sp7/Osterix* (B), and *Bglap/Osteocalcin* (C) in scramble control cells and *Nmur1* knockdown cells; Figure S3: Representative images of histological specimens from wild type control or *Nmur1* knockout; Table S1: Antibody array analyses using tibiae from wild type controls or *Nmu* knockout mice; Table S2: Antibody array analyses using tibiae from wild type controls or *Nmu* knockout mice; Table S3: Antibody array analyses using lysates from scramble control or *Nmur1*-KD cells +/− treatment with 1 µM NMU25 for four hours; Table S4: Antibody array analyses using lysates from scramble control or *Nmur1*-KD cells +/− treatment with 1 µM NMU25 for four hours.
