# Supplementary material for: NMUR1 in the NMU-Mediated Regulation of Bone Remodeling
Source: Life (Basel). 2021 Sep 29;11(10):1028. doi: 10.3390/life11101028 (PMC8538501; doi:10.3390/life11101028)
Supplement: Supplementary file 1 [file life-11-01028-s001.zip › life-1372215-supplementary.pdf]

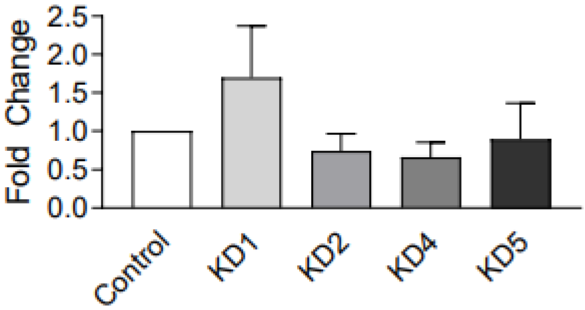

Figure S1. Expression of *Nmur1* in scramble control cells and putative *Nmur1* knockdown (KD) lines.

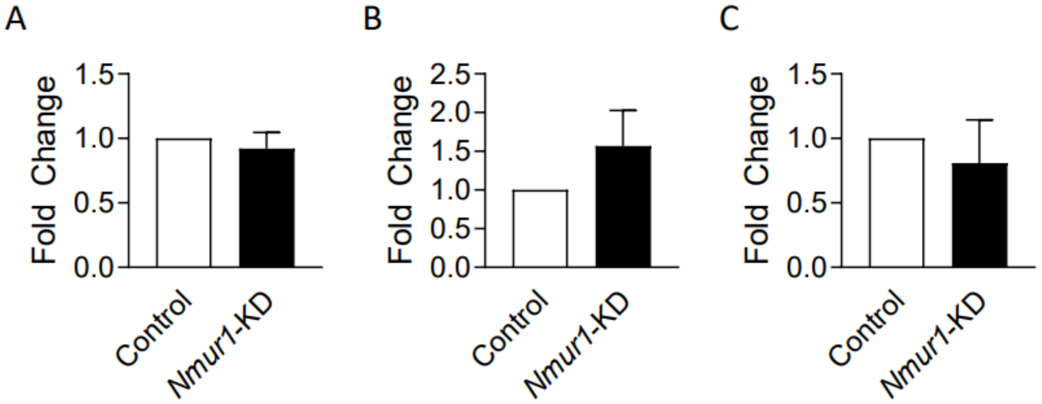

Figure S2. Expression of *Runx2* (**A**), *Sp7/Osterix* (**B**), and *Bglap/Osteocalcin* (**C**) in scramble control cells and *Nmur1* knockdown cells.

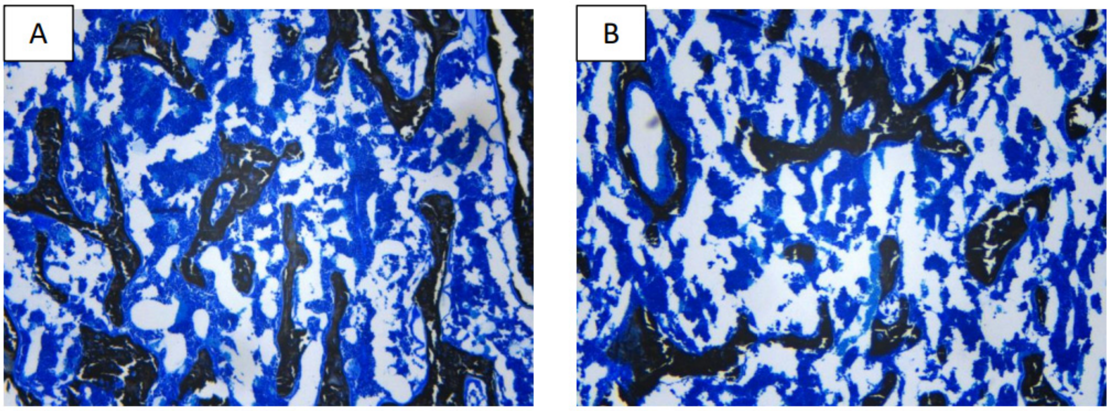

Figure S3. Representative images of histological specimens from wild type control or *Nmur1* knockout (**A**) and (**B**).
